# Emergency preparedness for those who care for infants in developed country contexts

**DOI:** 10.1186/1746-4358-6-16

**Published:** 2011-11-07

**Authors:** Karleen D Gribble, Nina J Berry

**Affiliations:** 1 School of Nursing and Midwifery, University of Western Sydney, Locked Bag 1797, Penrith NSW. 2751. Australia; 2 Centre for Health Initiatives, University of Wollongong, NSW, 2522. Australia

**Keywords:** disasters, emergencies, infant formula, artificial feeding, breastfeeding, emergency preparedness

## Abstract

Emergency management organisations recognise the vulnerability of infants in emergencies, even in developed countries. However, thus far, those who care for infants have not been provided with detailed information on what emergency preparedness entails. Emergency management authorities should provide those who care for infants with accurate and detailed information on the supplies necessary to care for them in an emergency, distinguishing between the needs of breastfed infants and the needs of formula fed infants. Those who care for formula fed infants should be provided with detailed information on the supplies necessary for an emergency preparedness kit and with information on how to prepare formula feeds in an emergency. An emergency preparedness kit for exclusively breastfed infants should include 100 nappies and 200 nappy wipes. The contents of an emergency preparedness for formula fed infants will vary depending upon whether ready-to-use liquid infant formula or powdered infant formula is used. If ready-to-use liquid infant formula is used, an emergency kit should include: 56 serves of ready-to-use liquid infant formula, 84 L water, storage container, metal knife, small bowl, 56 feeding bottles and teats/cups, 56 zip-lock plastic bags, 220 paper towels, detergent, 120 antiseptic wipes, 100 nappies and 200 nappy wipes. If powdered infant formula is used, an emergency preparedness kit should include: two 900 g tins powdered infant formula, 170 L drinking water, storage container, large cooking pot with lid, kettle, gas stove, box of matches/lighter, 14 kg liquid petroleum gas, measuring container, metal knife, metal tongs, feeding cup, 300 large sheets paper towel, detergent, 100 nappies and 200 nappy wipes. Great care with regards hygiene should be taken in the preparation of formula feeds. Child protection organisations should ensure that foster carers responsible for infants have the resources necessary to formula feed in the event of an emergency. Exclusive and continued breastfeeding should be promoted as an emergency preparedness activity by emergency management organisations as well as health authorities. The greater the proportion of infants exclusively breastfed when an emergency occurs, the more resilient the community, and the easier it will be to provide effective aid to the caregivers of formula fed infants.

## Introduction

The World Health Organization and UNICEF Global Strategy on Infant and Young Child Feeding recommends that infants be exclusively breastfed for the first six months of life and then continue to be breastfed, with the addition of complementary foods, for two years or more [1]. In an emergency situation, infants who are exclusively breastfed have their health and well being protected by the food, water and immune factors provided by breast milk. Breastfeeding also mitigates physiological responses to stress in both infants and their mothers, helping them to cope with the stress of being caught up in an emergency situation [2]. However, the majority of children in developed countries have their intake of breast milk partially or totally replaced by infant formula in their first year of life [e.g. [3-5]]. In developing country contexts, formula feeding is frequently fatal to the infant [6,7]. In developed countries, good infrastructure, including easy access to clean water, electricity and medical care, means that relatively few infants die as result of formula feeding [8]. The low mortality rate associated with formula feeding in developed countries contributes to the acceptability of partial or total formula feeding. However, when an emergency occurs, any one or all of the aforementioned resources that makes formula feeding relatively safe can be severely limited. Thus, in emergencies, those who care for formula fed infants may be faced with circumstances that make formula feeding extremely difficult and potentially very dangerous.

Reports from past emergencies have demonstrated the difficulties faced by those who care for formula fed infants in large-scale emergencies in developed countries (unless it is stated otherwise these reports were told to KG by health professionals who had worked with mothers during these emergencies). In the aftermath of Hurricane Katrina (New Orleans, 2005), dangerous feeding practices were widespread. Gruich [9] described finding a large number of infants being fed water alone, placing infants at risk of life threatening hyponatraemia [10]. News media [e.g. [11]] and health workers, reported infant deaths as a result of unavailability of suitable food. In Australia during the 2009 Black Saturday bushfires, wet nursing was applied when neither a mother nor the centre to which she was evacuated had infant formula or other milk available. During the 2011 Queensland flood, cleaning of feeding bottles in evacuation centres was problematic and in one centre mothers were advised to use small rocks to scarify and clean the inside of bottles. In all large scale emergencies in developed countries the need to ensure that formula fed infants have suitable food available to them has been a high priority of emergency management authorities.

While mothers who are exclusively breastfeeding are able to continue to provide food to their infants regardless of the stress they might be experiencing and their own access to food, emergency authorities have not necessarily understood this. During the 2007 wildfires in San Diego, California, one organisation was adamant that breastfeeding women who had been evacuated should accept the infant formula that they were distributing, regardless of mothers' insistence that they did not need it. The idea that breastfed infants are in a food insecure situation is reiterated by the US Red Cross and the Federal Emergency Management Agency, who suggest that breastfeeding mothers store infant formula in the event that they are unable to breastfeed in an emergency [12]. These expressions of formula feeding as providing food security and breastfeeding as unreliable are contrary to the experiences of individuals affected by emergencies and likely arise from cultural beliefs about infant feeding that are peculiar to developed country contexts.

Emergency management organisations recognise that the needs of infants necessitate particular care in emergencies. However, emergency preparedness materials generally provide only non-specific or incomplete information and do not distinguish between breastfed and formula fed infants. It is common for emergency preparedness materials to simply state that those who care for infants should take into account their special needs when packing a disaster preparedness kit or that those who care for infants should include infant formula in their disaster preparedness kits [13,14]. For example, the Emergency Management Australia publication, "Preparing for the Unexpected" includes in their emergency preparedness checklist "special needs for infants, the aged and people with disabilities," accompanying the text with a photograph of an infant feeding bottle full of milk. As far as the authors are aware, no emergency preparedness authority in a developed country mentions breastfeeding continuance as an emergency preparedness activity, nor details the requirements for formula feeding in an emergency. There is a need to improve emergency preparedness and the delivery of aid to the caregivers of infants in emergencies.

The purpose of this paper is to detail the supplies needed by the caregivers of breastfed and formula fed infants in an emergency situation where essential services such as electricity and clean water supplies are unavailable and to discuss some of the practicalities of caring for infants in emergencies. The amounts provided for each emergency item are based on the clinical experience of the authors', the author's trial of the procedures, and the manufacturer's instructions. This information is targeted at both emergency management organisations and individuals who care for infants. This paper also provides examples of the sorts of messages that emergency management organisations should provide to those who care for infants prior to an emergency, during an emergency and during the recovery period.

## Emergency kits for the caregivers of infants

### Breastfed infants

**Figure 1:**
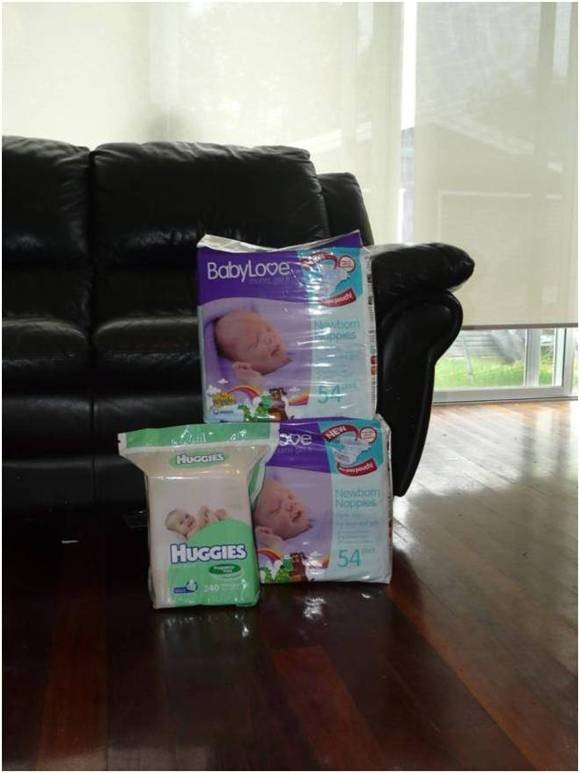
**Emergency supplies needed to care for an exclusively breastfed infant**.

In order to prepare for an emergency, mothers of exclusively breastfed infants do not need to store any food-related items for their babies. Exclusive breastfeeding could be considered an emergency preparedness activity. Thus, the only items necessary to store in preparation for an emergency if an infant is exclusively breastfed are nappies and nappy wipes. Approximately, one hundred disposal nappies (diapers) and two hundred nappy wipes would be sufficient for one week's emergency supply. In Australia, the cost of this emergency kit is approximately $50. Figure 1 shows an example of emergency supplies for the mother of an exclusively breastfed infant and emergency supplies are summarised in Table 1.

**Table 1:** Emergency supplies required for breastfed infants or for feeding infants using ready-to-use infant formula or powdered infant formula*

Exclusively breastfed infant	Exclusively formula fed infant with ready-to-use infant formula	Exclusively formula fed infant with powdered infant formula
100 nappies	56 single serves of ready-to-use infant formula	2 tins of infant formula
200 nappy wipes	84 L of water	170 L of water
	Large storage container	Large storage container
	Metal knife	Large cooking pot with a lid
	Small bowl	Kettle
	56 feeding bottles or cups	Gas stove
	56 zip-lock plastic bags	Box of matches or lighter
	220 sheets of paper towel	14 kg of liquid petroleum gas
	Detergent	Measuring container
	120 antiseptic wipes	Metal knife
	100 nappies	Metal tongs
	200 nappy wipes	Feeding cup
		300 sheets of paper towel
		Detergent
		100 nappies
		200 nappy wipes

Estimated cost $50	Estimated cost of consumables $550	Estimated cost of consumables $250

*Costs are presented in Australian dollars

Mothers who are exclusively breastfeeding during an emergency can continue to feed their infant as they did before the emergency occurred; no special actions are necessary.

### Formula fed infants

Emergency preparedness for exclusively formula fed infants involves storage of items necessary for feeding the infant. Different requirements exist dependent upon whether liquid ready-to-use infant formula or powdered infant formula is available or chosen for use in emergency preparedness. Whilst there is some overlap in what is needed and how supplies are used depending on whether ready-to-use liquid infant formula or powdered infant formula is stored, each instance is detailed individually for the sake of clarity.

#### Where ready-to-use liquid infant formula is available

**Figure 2:**
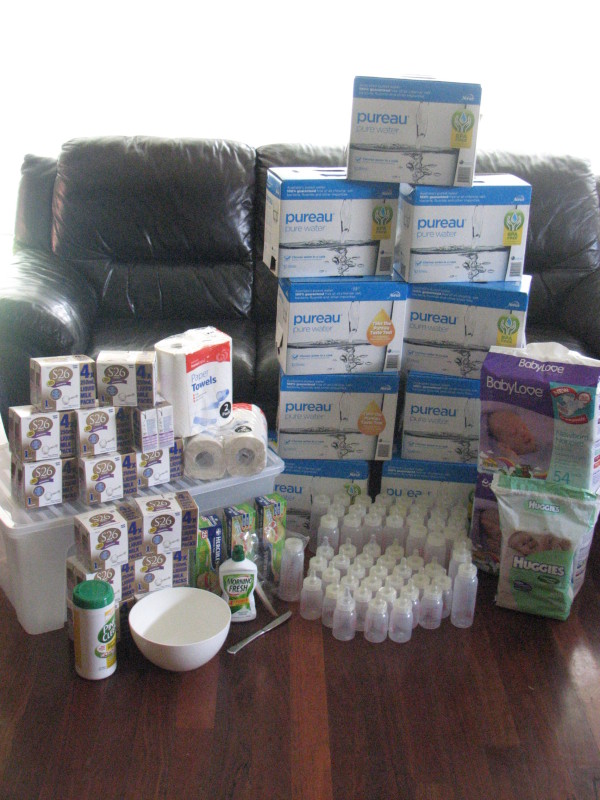
**Emergency supplies required to fully formula feed using ready-to-use infant formula**.

Supplies necessary to prepare to formula feed using ready-to-use liquid infant formula in an emergency include: infant formula, water, a storage container, a metal knife, a small bowl, feeding bottles and teats or cups, zip-lock plastic bags, paper towels, detergent and soap, and antiseptic wipes. Nappies and nappy wipes also need to be stored. The reason for each item and amounts required for one week's supply are described below. In Australia, the cost of the consumables in this emergency kit is approximately $550. Figure 2 shows an example of emergencies supplies needed to fully formula feed for one week using ready-to-use liquid infant formula and emergency supplies are summarised in Table 1.

1) Infant formula

The amount of infant formula required will depend upon the specifics of the product and the intake of the infant. It is important that sufficient packages of single serves of ready-to-use infant formula are stored because once a package is opened it must be used immediately and any left-over milk discarded. For infant formula available in Australia, one week's supply for an infant who is five months of age would usually be 56 packages of 250 ml ready-to-use infant formula. It is important to note that while follow-on infant formula (for infants older than six months of age) is not suitable for infants younger than six months of age, starter infant formula (for infants from zero to six months of age) can be fed to infants older than six months. Care should be taken to ensure that stored infant formula has not passed its expiry date.

2) Water

Approximately one and a half litres of water per feed is required for cleaning hands, the knife for cutting open the packages of ready-to-use infant formula and the preparation area. Where an infant is having eight feeds a day this translates to about 12 litres a day and about 84 litres for one week. Older infants may need slightly fewer feeds per day, especially if they are also eating complementary foods.

3) Storage container

A container in which the infant formula and preparation and cleaning implements can be stored is required. This storage container should have solid sides and a lid that seals well enough to protect the contents from dirt and insects. The lid should also be completely removable so that it can be turned over and act as a clean surface for the preparation of infant formula.

4) Metal knife

A metal knife which is sharp enough to cut open the packages of ready-to-use infant formula is required.

5) Small bowl

A small bowl in which the metal knife can be washed is needed.

6) Feeding bottles and teats or cups

Sufficient feeding bottles and teats or cups to feed the infant are required. In order to provide eight feeds a day, 56 bottles and teats, or cups, need to be stored. If bottles and teats are stored they should be thoroughly disinfected by washing in hot soapy water and then allowed to air dry before the teat is sealed inside the bottle and the bottle sealed in a plastic bag for storage. If cups are stored they should be similarly cleaned and dried before being sealed in a plastic bag for storage. Alternatively, new paper or plastic disposable cups can be stored. These feeding bottles and teats or cups are to be used as single use items and disposed of after a single use. As will be described, cleaning of feeding implements for formula feeding in an emergency requires a large amount of water and fuel as well as other resources and if reuse of feeding implements is desired these resources will need to be stored.

7) Zip-lock plastic bags

Fifty six zip-lock plastic bags that are large enough to contain each feeding bottle or cup are required.

8) Paper towels

Sufficient paper towels for drying hands and cleaning and drying the preparation surface and washing bowl are needed. Approximately 220 large sheets of paper towel are needed. A fabric drying cloth is not appropriate because it can act as a vector for contamination.

9) Detergent and soap

A bottle of detergent is necessary for washing hands, the knife and the preparation surface. Hard soap may also be stored if preferred for hand washing.

10) Antiseptic wipes

Antiseptic wipes are necessary to assist in ensuring that the knife used for cutting open the packages of ready-to-use infant formula and the preparation surface is disinfected. Approximately 120 wipes are required.

#### Instructions for using ready-to-use infant formula in an emergency

In the event of an emergency, the goods that have been stored should be used in the following way (these instructions are based on field experience with artificial feeding in emergency conditions).

1) Clean the preparation surface

It is important that the surface on which infant formula is prepared is clean. Since clean surfaces may be difficult to find in emergencies, "storing" a clean surface is desirable. The inside of the plastic or metal storage container provides a clean surface. The preparation surface should be made wet with water, squirted with detergent and then rubbed with paper towel. It should then be dried with another paper towel and finally wiped with an anti-bacterial wipe. The knife for cutting open the packages of infant formula should be cleaned and disinfected prior to first use (as described later).

2) Wash hands using soap and water

Washing hands is a critical step in the hygienic preparation of infant formula feeds. It is especially important in emergency situations where surfaces may be contaminated. Where clean water is in limited supply, hands should first be moistened by pouring water onto them. Then soap or detergent can be rubbed into a lather, with attention paid to ensure that all of the surfaces of the hands are rubbed clean. Finally, the soap and dirt can be rinsed off. The assistance of a second person to pour water onto hands may be helpful. Once clean, hands should be dried with clean paper towel.

3) Use the clean knife to cut open a package of ready-to-use infant formula

4) Pour the required amount of ready-to-use infant formula into the feeding bottle or cup

5) Feed the baby using the feeding bottle or feeding cup

Instructions on how to feed an infant using a cup are available from a number of sources [15-17]. In addition, lactation consultants and breastfeeding counsellors commonly have experience in assisting mothers to cup feed infants.

6) Discard any unused reconstituted infant formula within two hours

Alternatively, the formula can be consumed by older children or adult members of the family.

7) Discard the used feeding bottle or cup

Used feeding bottles or cups cannot be reused for feeding an infant. Should sufficient feeding bottles not be available for use, a reusable cup should be used which should be washed and sterilised between uses as described later in the description of procedure for formula feeding using powdered infant formula. Under no circumstances should care givers attempt to reuse feeding bottles in circumstances where water and power supplies are limited.

8) After feeding, hands should be washed as described above

9) Wash and disinfect the metal knife

Pour water and detergent into the washing bowl. Thoroughly wash the knife with soapy water and dry with paper towel before wiping with an antiseptic wipe and allowing it to air dry. The bowl should be rinsed in clean water and dried with paper towel.

10) Return all supplies (except the water) to the storage container and seal the lid.

#### Where powdered infant formula is available

**Figure 3:**
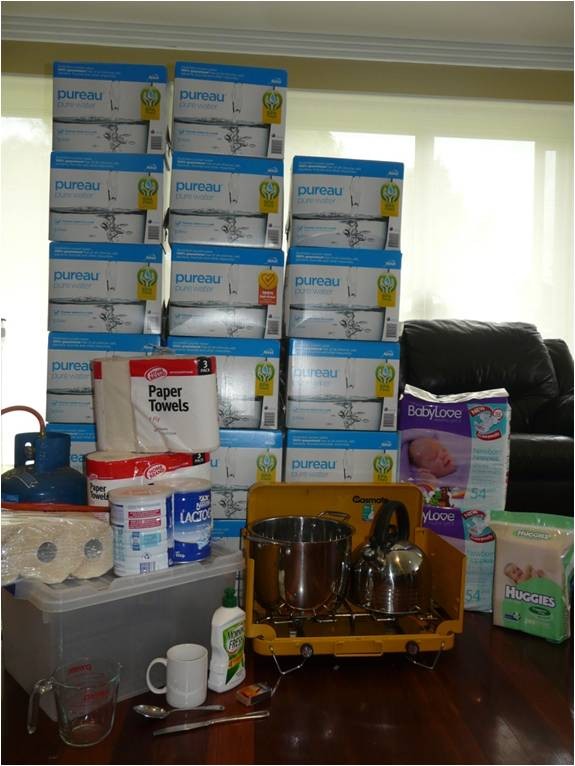
**Emergency supplies required to fully formula feed using powdered infant formula**.

Supplies necessary to prepare to formula feed using powdered infant formula in an emergency include: infant formula, water, a storage container, a large cooking pot with a lid, a kettle, a gas stove, matches or a lighter, liquid petroleum gas, a measuring container, a metal knife, metal tongs, a feeding cup, paper towels and detergent. Nappies and nappy wipes also need to be stored. The reason for each item and amounts required for one week's supply is described below. In Australia, the cost of the consumables in this emergency kit is approximately $250. Figure 3 shows an example of emergencies supplies needed to fully formula feed using powdered infant formula for one week and emergency supplies are summarised in Table 1.

1) Infant formula

The amount of infant formula required will depend upon the specifics of the product and the intake of the infant. For infant formula available in Australia, one week's supply for an infant of five months of age would usually be two 900 g tins of powdered infant formula. It is important to note that while follow-on infant formula (for infants older than six months of age) is not suitable for infants younger than six months of age, starter infant formula (for infants from zero to six months of age) can be fed to infants older than six months. Care should be taken to ensure that stored infant formula has not passed its expiry date.

2) Water

Approximately three litres of water per formula feed is required to reconstitute infant formula and clean hands, feeding and preparation implements and the preparation surface. Where an infant is having eight feeds a day this translates to about 24 L per day and about 170 L for one week. Older infants may need slightly fewer feeds per day, especially if they are also eating complementary foods. Bottled water may be used for reconstitution of powdered infant formula depending on the mineral concentration of the water. In order to be suitable for reconstituting infant formula water should contain less than 200 mg/L sodium, less than 250 mg/L sulphate and less than 50 mg/L nitrate [18], most bottled drinking water available in developed countries will meet these requirements.

3) Storage container

A storage container in which the infant formula and preparation and cleaning implements can be stored is required. This storage container should have solid sides and a lid that seals well enough to protect the contents from dirt and insects. The lid should also be completely removable so that it can be turned over to act as a clean surface for the preparation of infant formula. The storage container can also be used as a basin for washing preparation and feeding implements or alternatively, a separate container for washing can be stored.

4) Large cooking pot with a lid

This pot is to be used to boil water for sterilising preparation and feeding implements. It needs to be large enough to be able to completely submerge the measuring container, feeding cup, knife and spoon during the sterilisation process. It is always recommended that implements used to prepare and feed infant formula be sterilised [19], however the need for this process is magnified in emergency situations where surfaces can be easily contaminated. This cooking pot should not be used for purposes other than sterilising (for example, it should not be used for cooking food).

5) Kettle

A kettle which can be used to boil water on the gas stove for reconstituting infant formula is needed. The kettle is used to heat water for reconstitution of the infant formula and for washing feeding and preparation implements. Heating water for reconstitution of infant formula is necessary because bottled water is not sterile and because powdered infant formula is commonly contaminated with pathogenic bacteria [19].

6) Gas stove

A gas stove for the heating of the water needed to reconstitute infant formula, clean and sterilise feeding and preparation implements and clean surfaces is required. The gas stove should be large enough to safely heat water in the large cooking pot and kettle.

7) Sufficient matches or a lighter

Matches or a lighter are necessary for the lighting of the gas stove. A lighter may be preferable to matches because of the possibility of matches becoming unusable if they come in contact with water. Alternatively, waterproof matches may be stored.

8) Liquid petroleum gas

Approximately 14 kg of liquefied petroleum gas (LPG) is required to heat water for reconstituting infant formula and for washing and sterilising. This amount is based on the following calculation. Energy required to heat 24 L of water from 15°C to boiling temperature at sea level, that is 100°C, is given by the equation Q = m cp (100-15)K (where Q is the amount of energy required, m is the mass of water, cp is the heat capacity of water, that is cp = 4.817 kJ/(kg K) and the numbers in parenthesis represent the temperature difference). Then, Q = 23.5 kg × 4.817 kJ/(kg K) × 85 K = 9621.96 kJ per day of energy required. LPG has a specific caloric value of 46.1 MJ/kg. However, this energy cannot be perfectly used to heat water, it is estimated that 90% of the energy will be lost. Thus the estimate of the amount of LPG per day required is 2 kg per day or 14 kg for one week. If the gas stove is to be used for cooking, additional fuel will need to be stored.

9) Measuring container

A heatproof, accurate measuring container is required for measuring the water needed to reconstitute the infant formula and for mixing the infant formula. Infant feeding bottles cannot be used for reconstitution of infant formula because (as will be discussed) they are too difficult to clean.

10) Metal knife

A metal knife is needed for levelling measured scoops of powdered infant formula.

11) Metal spoon.

A metal spoon is needed to mix the powdered infant formula and water.

12) Metal tongs

A pair of metal tongs is needed to remove sterilised preparation and feeding implements from hot water.

13) Feeding cup

An open ceramic or metal cup is needed for feeding the infant. The cup should be shallow enough for the corners to be able to be easily reached by fingers during cleaning. Neither a feeding bottle nor a spouted cup is appropriate for feeding an infant in an emergency because of the difficulties associated with cleaning them in the absence of running water and electricity/gas plumbing. Infant feeding bottles are particularly difficult to clean adequately even in the best of circumstances; a study of bottle cleanliness in the UK found that more than 60% of "cleaned" bottles sampled were contaminated with bacteria at a level such that they could not be considered as clean [20]. In an emergency situation, where water may be limited and surfaces contaminated, cleaning feeding bottles and teats adequately may be impossible, placing infants at a heightened risk of infectious disease. Cup feeding is the recommended practice when formula feeding is necessary in resource poor settings, including in emergencies [16,21-23].

14) Paper towels

Sufficient paper towels for drying hands, the metal knife and spoon and the cooking pot and wiping the preparation surface are needed. Approximately 300 large sheets of paper towel are needed. A fabric drying cloth is not appropriate because it can act as a vector for contamination.

15) Detergent

A bottle of detergent is necessary for washing hands, the feeding and preparation implements and the preparation surface. Hard soap may also be stored if preferred for hand washing. Anti-bacterial wipes may also be stored for wiping down the infant formula preparation surface.

16) Nappies

One hundred disposable nappies.

17) Nappy wipes

Two hundred nappy wipes.

#### Instructions for using powdered infant formula in an emergency

In the event of an emergency, the goods that have been stored should be used in the following way (these instructions are based on those provided in the publication Safe Preparation and Handling of Powdered Infant Formula [19]).

1) Clean the preparation surface

It is important that the surface on which infant formula is prepared is clean. Since clean surfaces may be difficult to find in emergencies, "storing" a clean surface is desirable. The inside of the plastic or metal storage container provides a clean surface. The preparation surface should be made wet with hot water, squirted with detergent and then rubbed with paper towel. It should then be dried with another paper towel. The surface may also be wiped with anti-bacterial wipes after washing with detergent and water. Feeding and preparation implements should be cleaned and sterilised prior to first use (as described later).

2) Wash hands using soap and water

Washing hands is a critical step in the hygienic preparation of infant formula. It is especially important in emergency situations where surfaces may be contaminated. Where clean water is in limited supply, hands should first be moistened by pouring water onto them. Then soap or detergent can be rubbed into a lather, with attention paid to ensure that all of the surfaces of the hands are rubbed clean. Finally, the soap and dirt can be rinsed off. The assistance of a second person to pour water onto hands may be helpful. Once clean, hands should be dried with clean paper towel.

3) Boil water for reconstituting powdered infant formula

Boil a sufficient quantity of clean water using the kettle and allow the water to cool for five minutes.

4) Measure the required amount of water using measuring container (sterilised after the last feed)

Place the water into the feeding cup (sterilised after the last feed).

5) Measure precisely the required amount of powdered infant formula

Dip the scoop into the powdered infant formula, ensuring that it is filled above the rim. Do not press or pack the powder into the scoop. Level the measuring scoop by running the back of a knife across the rim of the scoop, allowing the excess to be returned to the tin. Add the powdered infant formula to the hot water in the feeding cup.

6) Stir to mix with the metal spoon (sterilised after the last feed).

7) Cool the feed

Ensure that the reconstituted infant formula has cooled sufficiently before feeding to the infant. If sufficient water is available, the cup containing the reconstituted infant formula can be placed into a container of clean, cool water to speed cooling. Alternately, vigorous stirring will speed cooling. The temperature of the feed should be tested by stirring and then dropping a small amount of the reconstituted milk onto a carer's inner forearm where it should no longer feel warm.

8) Feed the baby using the feeding cup

Instructions on how to feed an infant using a cup are available from a number of sources [15-17]. In addition, lactation consultants and breastfeeding counsellors commonly have experience in assisting mothers to cup feed infants.

9) Discard any unused reconstituted infant formula within two hours.

Alternatively, the formula can be consumed by older children or adult members of the household.

10) After feeding, before cleaning and sterilising preparation and feeding implements, hands should be washed as described above.

11) Wash and sterilise preparation and feeding implements thoroughly

Boil sufficient water to wash preparation and feeding implements. Place a sufficient quantity of this water and detergent into the storage container or a basin reserved for this purpose. Great care should be taken to ensure that corners are thoroughly cleaned. Preparation and feeding implements should then be rinsed in clean water and dried with paper towel before storage in storage container (which will also need to be cleaned and dried if it has been used for washing).

12) Sterilise preparation and feeding implements in preparation for the next feed

Preparation and storage implements should be completely submerged in water in the large cooking pot and the water brought to the boil on the gas stove. Care should be taken to ensure that there are no trapped air bubbles. When the water is at a rolling boil the gas may be turned off and the implements left in the water in the lidded pot until they are needed. Implements should be removed from the pot just before they are to be used using the clean metal tongs or, if this is not practical, returned to the storage container for storage.

13) Return everything except the water, cooking stove and gas bottle to the storage container and seal the lid.

## Preparedness for infants older than six months

Infants who are older than six months of age are less vulnerable than younger babies. They are able to eat solid foods and can safely consume some water. Their immune systems are also more mature. Supplies required by mothers of breastfed infants older than six months should include sufficient nappies and wipes and also some complementary foods. However, should a mother not have appropriate complementary foods stored, her infant can revert to exclusive breastfeeding without harm, as her milk supply will increase in response to increased demand. Those who care for formula fed infants older than six months of age should also store complementary foods and the goods previously described as necessary for formula feeding, with an adjustment based on the intake of the child. Where appropriate complementary foods are available, the amount of complementary food can be increased if infant formula is in limited supply (even replacing infant formula entirely for a period of time). Infants older than six months can also be given water to prevent dehydration.

## Providing aid to the caregivers of infants

In a large scale emergency, providing aid to the caregivers of infants, particularly infants who are formula fed, should be a priority. Infants, particularly the very young, who do not have access to appropriate food can become very sick and even die within days. However, the distribution of supplies needed to formula feed should be handled by suitably experienced health workers (not food distribution workers) who are able to provide an assessment of the needs of the infant and provide education on how to prepare formula feeds using the available resources. Should ready-to-use single serves of infant formula with disposable bottles and teats be available, this can be distributed in preference to powdered infant formula and other resources (it should be made absolutely clear that any left-over formula should be discarded after each feed and that bottles cannot be reused). Wherever possible, women who are mixed breastfeeding/formula feeding should be encouraged to avoid using infant formula - and assisted to provide for all of their babies' nutritional needs by breastfeeding them very frequently (as often as hourly should be expected). Enormous care should be taken to ensure that infant formula is not distributed to breastfeeding mothers; previous experience has shown that where infant formula is given to breastfeeding mothers it is frequently used and results in increased rates of diarrhoeal illness in infants [24]. It should also be considered that mothers of young, fully formula fed infants may still be lactating and be able to reinitiate breastfeeding fairly easily. In the stressful circumstances of an emergency, it cannot be assumed that mothers will think of reinitiating breastfeeding. One of the infant deaths following Hurricane Katrina was a three week old fully formula fed baby who had been stranded on a roof for five days after the Hurricane with his mother who did not have formula to feed him. The baby was alive when rescued but later died in hospital. A medical assessment of the infant's mother revealed that she had breasts engorged with milk, however the possibility of initiating breastfeeding had not occurred to her.

It should not be taken for granted that mothers who are breastfeeding do not need assistance; breastfeeding mothers need support to continue breastfeeding, especially if they are experiencing difficulties. Emergency authorities should access their local mother-to-mother breastfeeding support organisation as a resource to both emergency workers and to mothers. Mother-to-mother support organisations, while specialising in assisting breastfeeding mothers, will also be able to help formula feeding mothers who wish to avoid using infant formula by re-establishing or increasing breastfeeding. They are also often a repository of expertise on the cup feeding of infants. Emergency organisations should ensure that they have policies and appropriate training in place to enable good practice in the delivery of aid to those who care for infants.

## Caring for formula fed infants when resources to formula feed are not available

In the event that there are formula fed infants, but not the resources necessary to formula feed, the following options may be possible. A breastfeeding mother may be available to share breast milk with the infant, either directly from the breast or fed expressed milk in a cup. It should be understood that once breast milk is expressed it has many of the hazards associated with infant formula. Thus, breast milk should only be expressed via hand (there is no place for breast pumps in emergency response because of the difficulties of cleaning the pumps) and expressed breast milk should be fed using a disposable cup (never a bottle). If fresh, commercial cows' milk is available, it may be made suitable as a short term replacement for infant formula for infants under six months by the addition of water and sugar (to 100 ml of boiled milk add 50 mls of water and two level teaspoons of sugar) [17]. It is important that infants under six months of age not be given water to drink because of the risk of hyponatraemia, nor undiluted cows' milk because of the risk of kidney damage.

## Emergency messages

Prior to emergencies, during emergencies and in the emergency recovery period, emergency management organisations should distribute messages targeted at those who care for infants.

### Prior to emergencies

Exclusive and continued breastfeeding should be promoted by emergency management organisations as an emergency preparedness activity that provides infants with a safe and secure food and water supply and protection from infection. In areas with seasonal emergencies, communications might include a message such as, *"mothers considering ceasing breastfeeding should consider waiting until after the [bushfire/wildfire/cyclone/hurricane/typhoon/flooding/snowstorm] season." *In areas susceptible to emergencies such as earthquakes and volcanic eruptions, the importance of breastfeeding as a protective behaviour should similarly be promoted. The caregivers of formula fed infants should be provided with information on all of the resources necessary to formula feed in an emergency. Emergency management agencies should avoid at all costs communicating the idea that emergency preparedness for the caregivers of formula fed infants simply involves storing some additional infant formula. In locations susceptible to emergencies, health professionals should routinely discuss emergency preparedness with those who are caring for infants. Those involved in the delivery of aid should be provided with appropriate training on infant and young child feeding in emergencies in order to be able to appropriately manage and deliver aid to the caregivers of infants.

### During emergencies

Infants are at greatest risk during the acute phase of emergencies. Messages should be targeted to the mothers of breastfed infants outlining the importance of exclusive and continued breastfeeding, the ways in which mothers can increase their milk supply and details of where mothers can obtain assistance if they are experiencing breastfeeding difficulties. Breastfeeding women should be exhorted to avoid starting to use infant formula during the acute emergency and recovery. Messages should also be targeted to those who care for formula fed infants providing detail on how infant formula should be prepared and delivered in emergencies, the importance of not giving young infants water and details of where caregivers can obtain supplies and assistance with formula feeding.

Emergency management agencies should communicate to the general public the message that while infants are vulnerable and require assistance in emergencies, they require a specific, targeted and supported type of aid. It should be made clear that donations of infant formula are not appropriate and can be harmful. It has been repeatedly found that in the wake of an emergency, large quantities of donations of infant formula arrive. These donations cause logistical difficulties and are often distributed inappropriately (for example given to breastfeeding women or to caregivers of formula fed infants without the other supplies necessary to formula feed [e.g. [24]]). It is generally accepted that donations of infant formula (including from infant formula manufacturers who have historically used emergencies as a marketing opportunity [e.g. [25]]) should be actively discouraged in all emergencies. On the contrary, monetary donations will enable the delivery of appropriate aid to infants. Where infant formula is purchased by aid organisations there is a greater likelihood of the distribution being carefully managed, which benefits both breastfed and formula fed infants.

### During the emergency recovery period

The recovery period of an emergency is often a time when the risk of contracting an infectious disease increases. During the recovery period, messages should be targeted to the caregivers of infants highlighting the importance of exclusive and continued breastfeeding and the importance of maintaining cleanliness and sterilisation in the preparation of infant formula in order to prevent infectious diseases such as gastroenteritis and respiratory disease.

Further information on infant and young child feeding and the media is available in the resources of the Infant and Young Child Feeding in Emergencies Core Group [26]. Examples of media messages suitable at each stage of an emergency are presented in Table 2.

**Table 2:** Examples of media messages on infant feeding issues at different stages of an emergency

Stage of emergency	Messages
Prior to the emergency	*Mothers considering ceasing breastfeeding should consider waiting until after the [bushfire/wildfire/cyclone/hurricane/typhoon/flooding/snowstorm] season*.
	*Community members should support mothers to exclusively breastfeed their infants in preparation for the emergency season*.
	*Those caring for infants who are formula fed should store the supplies necessary to formula feed in situations where power and water supplies are disrupted. These supplies include [list as per outlined in this paper]*.

During the emergency and the emergency recovery period	*As a result of this emergency, infants are at risk of serious illnesses like diarrhoea and respiratory infections. Mothers who are breastfeeding should continue breastfeeding, because it gives the baby clean water and food and protects them from infection. If your infant is under six months of age, do not give them any other food or liquid because this will make them vulnerable to infection*.
	*Breastfeeding protects babies during emergencies. Stress does not affect mothers' milk supply. Women who are experiencing difficulties with breastfeeding can find assistance at [insert location or phone number where mothers can access health professional or peer support for breastfeeding]*.
	*Breastfeeding protects infants from infection during this emergency. Mothers can increase their milk supply by breastfeeding more frequently*.
	*Infants who are formula fed are at serious risk in this emergency. Those who are caring for formula fed infants should take great care to ensure that water used in formula feeding is clean and that all feeding implements are thoroughly cleaned and sterilised before each feed is prepared*.
	*During this emergency infant feeding bottles may not be able to be properly cleaned because water and power are scarce. Feeding bottles that are not properly cleaned can harbour disease. Those who are caring for formula fed infants are advised to use an easily cleanable cup for feeding*.
	*Support for those who are caring for formula fed infants can be obtained at [insert location or phone number where care givers can access health professional or peer support for formula feeding]*.
	*People who want to help babies in this emergency can do so by donating money to [insert organisations which are delivering aid to infants]. Please do not donate goods (including infant formula)*.
	*Breastfeeding women who are given donated infant formula should not give it to their babies. Giving an exclusively breastfed baby infant formula can make them vulnerable to infection for weeks. Exclusive breastfeeding protects babies from infection*.
	*Infants younger than six months of age should not be given water to drink. It can make them extremely ill and may even be fatal*.
	*Infants and young children are at risk in this emergency. Friends, relatives and neighbours can help babies and young children by assisting their caregivers in practical ways*.

## Practicalities of formula feeding in emergencies

The quantity of supplies required to formula feed in an emergency, as well as the process of formula feeding in an emergency as described here, raises the question of the feasibility of preparing for an emergency if caring for a formula fed infant. Certainly the volume of supplies means that if the emergency necessitates leaving the home that it may not be possible to transport the supplies. Thus, whenever an emergency occurs in a developed country context it should be assumed that there will be a significant number of formula fed infants at immediate risk and identification of these infants and provision of necessary supplies should be of the highest priority.

Even where those who care for formula fed infants have the necessary supplies, the difficulty of the process of formula feeding itself may leave little room for other necessary activities and replenishment of supplies may also be a problem. Should essential services such as electricity and piped clean water not become available within a short time, and ready-to-use single serves of infant formula (with disposable bottles and teats) not be available, evacuation of formula fed infants and those who care for them should be considered.

## Conclusions

Emergency management organisations recognise the vulnerability of infants in emergencies, even in developed country contexts. However, thus far, the caregivers of infants have not been provided with detailed information on what emergency preparedness entails. Emergency management authorities should provide the caregivers of infants with accurate and detailed information on the supplies necessary to care for infants in an emergency, distinguishing between the needs of breastfed and the needs of formula fed infants. The caregivers of formula fed infants should be provided with information on how to prepare formula feeds in an emergency. Child protection organisations should ensure that foster carers responsible for infants have the resources necessary to formula feed in the event of an emergency. Exclusive and continued breastfeeding should be promoted as an emergency preparedness activity by emergency management organisations as well as health authorities. The greater the proportion of infants exclusively breastfed when an emergency occurs, the more resilient the community is and the easier it will be to provide effective aid to the caregivers of formula fed infants.

## Competing interests

The authors declare that they have no competing interests.

## Authors' contributions

KDG and NJB conceived of the paper and drafted the manuscript together. Both authors read and approved the final manuscript.

## Authors' interests

KDG is researcher with an interest in infant feeding issues. She is a member of the Infant and Young Child Feeding in Emergencies Core Group and has acted as a consultant to emergency organisations including UNICEF and the Emergency Nutrition Network in the development of training and policy materials on infant feeding in emergencies for the past five years.

NJB has a PhD in public health and holds nationally recognised qualifications in infant feeding counselling. In 2009 she coordinated breastfeeding counselling and artificial feeding programs following Cyclone Nargis in Myanmar for Save the Children UK.
